# Emergency treatment of splenic injury in a novel mobile minimally invasive interventional shelter following disaster: a feasibility study

**DOI:** 10.1186/s13049-014-0044-4

**Published:** 2014-08-09

**Authors:** Tianming Yao, Jingjing Rong, Ming Liang, Jingyang Sun, Fengqi Xuan, Lijun Zhao, Xiaozeng Wang, Fei Li, Geng Wang, Yaling Han

**Affiliations:** 1 Department of Cardiology, The general hospital of Shenyang Military Region, Shenyang 110016, China; 2 Third Military Medical University, Chongqing 400038, China

**Keywords:** Splenic injury, Emergency, Interventional therapy, Shelter

## Abstract

**Background:**

There has been an increase in natural disasters in recent years, which leads to a great number of injuries and deaths. It still remains an unsolved problem to treat patients with vascular injury of solid organs effectively following natural disasters, but on-spot emergency interventional transcatheter arterial embolization (TAE) has been highly recommended to cure serious vascular injury of solid organs nowadays. Spleen is the most vulnerable abdominal organ, severe arterial hemorrhage of which can cause death if untreated timely. In this research, we aimed to study the possibility of performing emergency surgical intervention in mobile minimally invasive interventional shelter for splenic injury in the case of natural disasters.

**Methods:**

First, the mobile minimally invasive interventional shelter was unfolded in the field, and then disinfection and preoperative preparation were performed immediately. Eight large animal models of splenic injury were created, and angiograms were performed using a digital subtraction angiography machine in the mobile minimally invasive interventional shelter, and then the hemostatic embolizations of injured splenic artery were performed following the established convention of rapid intervention therapy. The operating time was recorded, and the survival condition and postoperative complications were observed for two weeks.

**Results and discussion:**

The average time of unfolding the shelter, and performing disinfection and preoperative preparation was 33 ± 7 min. The number of colonies in the sterilized shelter body was 86 ± 13 cfu/m^3^. The average TAE time was 31 ± 7 min. All the hemostatic embolizations of splenic injury were performed successfully in the mobile minimally invasive interventional shelter during the operation. A pseudoaneurysm was found in an animal model using angiography two weeks after the operation. The primary clinical success rate of embolization was 87.5%. The two-week survival rate in all animal models of splenic injury was 100%.

**Conclusions:**

Our findings in the current study demonstrate that the mobile minimally invasive interventional shelter can be adapted to the field perfectly and complete emergency surgical intervention for splenic injury efficiently and safely. Therefore, on-spot emergency interventional TAE for vascular injury of solid organs (e.g. spleen) in mobile minimally invasive interventional shelter is available and effective.

## Background

When the human body suffers from a bump, a crush or some other fatal injuries in a catastrophe, the spleen is the most vulnerable organ in the abdomen, because it is a superficial and frangible organ [1]. Splenic injury accounts for 40%-55% of the abdominal traumas in the emergency treatment [2], and the severe arterial hemorrhage secondary to the splenic injury is considered the most common and serious complication that can lead to death in such a case [3],[4]. Therefore, effective hemostasis is essential in managing splenic artery injury. In the past, patients suffering from splenic injury under normal conditions always had to undergo emergency surgical operations to stanch bleeding as soon as possible [5]. Once severe hemorrhage occurs, emergent evacuation and effective surgery should be considered. This kind of operation must be performed in an operating room and is time consuming. In addition, it is always delayed by the poor treatment conditions or jammed evacuation routes following natural disasters. Once the opportunity for effective treatment is missed, the life of the casualty will be threatened, leading to a poor prognosis. Furthermore, there is a growing controversy over the immunodeficiency following splenectomy [3],[6]. Therefore, on-spot emergent interventional transcatheter arterial embolization (TAE) devices are highly recommended for treating patients with splenic injury [7].

For on-spot emergent interventional TAE, which can be appropriate for post-disaster treatment, mobile angiographic apparatuses are quite necessary. Therefore, we developed a novel minimally invasive interventional shelter, which is equipped with all required medical equipment in interventional treatment, including a new medium angiographic system. The shelter is highly mobile and is applicable to all terrains. The level of interventional diagnosis and therapy of this newly developed shelter is equal to that of the interventional system used in a first-rate hospital. However, the cost of building a mobile shelter is only one-half that of building a standard catheterization laboratory. Its use-cost is mainly made up of the cost of transport, medical materials (such as catheters, contrast agents, embolic agents and other materials) and maintenance.

In this research, we studied the feasibility of performing on-spot emergency interventional TAE in the patients with splenic injury in the mobile minimally invasive interventional shelter during natural disasters. We aim to treat the patients with severe splenic injury timely, and to avoid the life-threatening hemorrhage.

## Methods

### 1. Shelter unfolding and preoperative preparation

**Figure 1:**
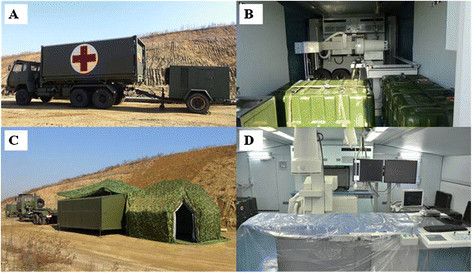
**Different states of the novel mobile minimally invasive interventional shelter. A**. transport state of the shelter; **B**. the interiors of the shelter; **C**. unfolded state of the shelter; **D**. working state of the shelter.

The novel mobile minimally invasive interventional shelter is a standard bilaterally-extending lab which can be carried by truck (Figure 1), transport plane, railway or cargo ship to the area of interest. The shelter is composed of a roof, a floor and two-side plates which can be extended or withdrawn by a controllable hydraulic pressure system, thus enabling the mobile unit to work on most ground conditions, fit all weather conditions, and easy and fast to transport. The cabin volume during folding (transport dimensions, Figure 1A) and unfolding (working dimensions, Figure 1C) is 6.05 m × 2.4 m × 2.4 m and 6.05 m × 6.3 m × 2.4 m, respectively. It is equipped with all necessary medical instruments for emergency interventional diagnosis and treatment, including a new medium C-arm radiographic system (maximum current: 200 mA, maximum voltage: 120 kV), an electrocardiography machine, a defibrillation monitor, an invasive blood pressure monitor, a high-pressure injector, an intra-aortic balloon pump (IABP), a portable B-mode ultrasound scanner and various modular interventional consumables. In addition, a portable anesthesia machine and common surgical kits are included. All the medical equipment was designed to be suitable for transportation. Besides heating facilities, air filtration and purification equipment have been added into the shelter to build proper operating environment. Meanwhile, a mobile telemedicine system allows the mobile unit to communicate with medical centre for remote technical support.

In this research, the novel mobile minimally invasive interventional shelter was unfolded for 4 times in open fields in Northeast of China in spring. The outdoor temperature was 6-12°C. The wind speed was 0 -15 m/s and the relative humidity was ≤55%. Two interventional radiologists, two nurses, a technician and a driver were needed as staff in the whole process. The air conditioner was then started by the technician to clean the air and lower the temperature to 25-27°C (In winter, air warmer is recommended) according to the requirements of operation. Then the disinfections were performed, including dust cleaning, laminar air flow purifying, spraying and wiping disinfection, and ultraviolet disinfection. The disinfection efficacy was tested by a natural precipitation method [8]. Five 9 cm agar plates were exposed at the corners, 1.5 m above the ground in the shelter body for 5 min. And then, the agar plates were transferred at 37°C for bacterial cultures. The number of colonies in the shelter body was calculated.

**Figure 2:**
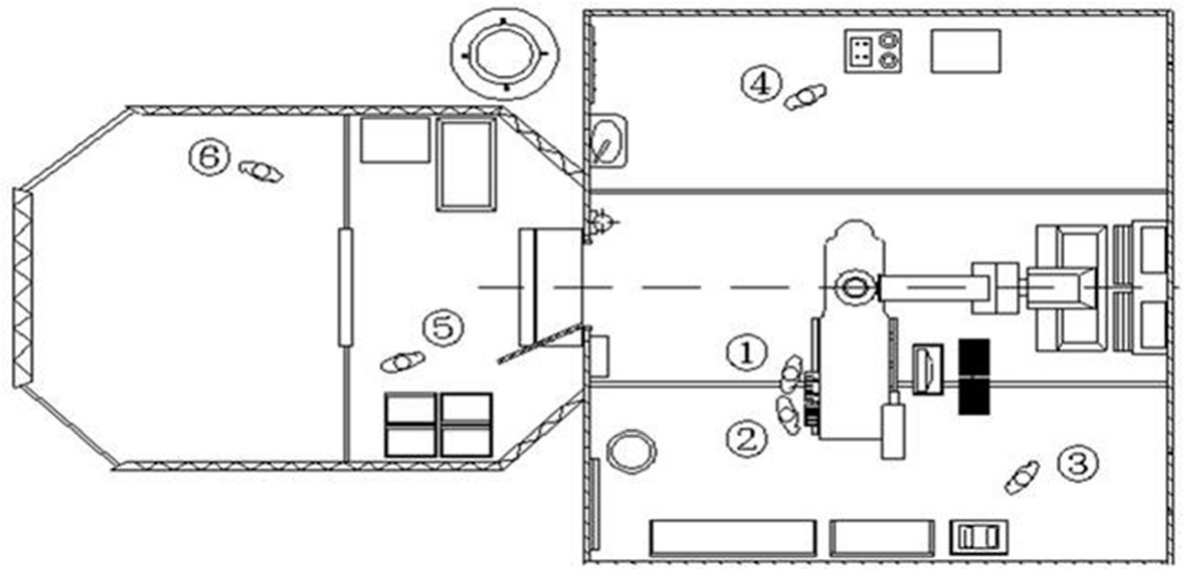
**Positioning sketch of the staff in working field interventional cabin. ① **performer **② **assistant **③ **nurse **④ **technician **⑤ **head nurse **⑥ **triage doctor.

The intervention instrument package and apparatus were put on the instrument tables from the transport containers by the nurse, and the intervention materials (including embolic material, gelatin sponge, 18# puncture needle, angiography catheter, loach guide wire, 9# Swartz sheath, 6 F vein sheath, contrast agent, heparin and physiological saline) were prepared. The pre-prepared pieces (1 mm^2^) of gelatin sponge were used as embolic materials before operation and saturated into contrast agent. Meanwhile, the interventional devices were started and checked by the technician to assure that they were in good working order. The technician was in charge of the light, angiography machine preheating and booting up the image-processing computer workstation. Finally, the interventional radiologists (including performer and assistant), nurses and technician were in their positions, ready to perform interventional embolization (Figure 2). The time from arriving at the spot to accomplishing preoperative preparation was recorded.

### 2. Animals and modeling splenic injury

The animal models were provided by the experimental animal center of Shenyang Northern Hospital and the experiment was supervised and authorized by the Ethics Committee of Shenyang Northern Hospital. Eight healthy mongrel dogs (male or female weighting between 25-30 kg) were chosen (2 for each embolization operation) and not feed for 24 h before the experiment, and then were given loading dose of aspirin (300 mg) and clopidogrel (600 mg) to eliminate thrombosis associated with interventional catheter or autologous clot and avoid impacting the effect of embolization therapy. The animal model was then fixed on the operation table for angiography. The airway was maintained with endotracheal intubation. Anesthesia was then induced by intravenous injection of propofol (3 mg /kg) and maintained by a syringe pump [10 mg /(kg · h)] during the experiment, and the heart rate and rhythm was recorded by ECG monitor.

**Figure 3:**
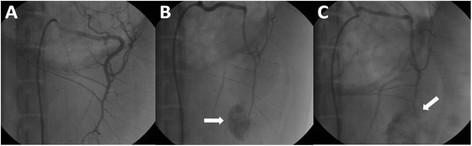
**The angiographic comparison of normal spleen, bleeding of injured splenic artery and hemostasis after embolization. A**. Angiographic image of normal spleen. **B**. Splenic injury model (white arrow shows the retention of contrast agent). **C**. Successful embolization of splenic artery (white arrow shows the distal portion of damaged artery is embolized and the hemorrhage is controlled).

The bilateral groin regions of the animal were sterilized and local anesthesia was achieved with 2% lidocaine. An 18# puncture needle (Seldinger technique) was used to puncture the right femoral artery, and then a 6 F vein sheath was inserted into the puncture needle. 5000-10000 U of heparin and physiological saline was injected into the side tube and 1000-2000 U was added per hour as supplement. Under the guidance of loach guide wire, the angiography catheter was inserted along the sheath to perform splenic artery angiography (Figure 3A). After removing the angiography catheter, the Swartz sheath was inserted into the splenic artery and was pushed forward to pierce the splenic artery for making a damaged model. Later, the sheath was drawn out and the puncture point was dressed with appropriate pressure. Splenic artery injury was thus justified by the leakage of contrast agent shown on the screen (Figure 3B).

### 3. Urgent arterial embolization of splenic injury

20 min after the splenic injury model was created, the 18# puncture (Seldinger technique) needle was used to puncture the left femoral artery. Angiographic intervention was performed again to observe the consequent image leakage of contrast agent. We pushed forward the angiography catheter along the guide wire to the main splenic artery and locate the tip of the catheter into the proximal main splenic artery under the supervision of angiography. The contrast agent, which contained gelatin sponge pieces, was injected into the main splenic artery through angiography catheter. Changes in the blood flow velocity in the main splenic artery were observed to determine whether to stop the sponge pieces injection. Once the embolization succeeded, the injection should be halted, with the operating time recorded. It was advisable to check the effect of embolization though angiography within 10 min (Figure 3C). Finally, the puncture point was dressed with appropriate pressure and animals were sent back to the experimental animal center.

### 4. Postoperative monitoring

The survival condition of animal models was observed and angiography was performed 2 weeks after hemostatic embolization to find out postoperative complications.

## Results

The time of preoperative preparation (unfolding the shelter, disinfection, etc.) in the 4 experiments was 29–38 (33 ± 5) min. The number of colonies in the sterilized shelter body was 69–117 (88 ± 18) cfu/m^3^. The image quality of medium C-arm met the requirements of interventional operation and the splenic arteries were displayed distinctly (Figure 3). The 8 animal models of splenic injury were created successfully (Figure 3B). The urgent hemostatic embolizations of 8 splenic injuries were performed successfully (Figure 3C) and all the equipments in the mobile minimally invasive interventional shelter worked properly. The TAE time was 23–42 (31 ± 7) min. Two weeks after operation, a pseudoaneurysm was found in an animal model using angiography and the primary clinical success rate of embolization was 87.5%. The two-week survival rate of all animal models of splenic injury which received urgent arterial embolization was 100%.

## Discussion

The mobile minimally invasive interventional shelter is developed to meet the demand of medical aid after serious natural disasters and it can be used for the minimally invasive interventional diagnosis and treatment. TAE of parenchyma organ injury (such as splenic injury in this research) is one of the primary functions of the mobile unit. It is also feasible to use it for other interventional procedures on the spot following natural disasters, for example, emergency treatment of coronary heart diseases by coronary artery angiography and stenting [7], internal iliac artery rupture by balloon occlusion, and so on. In addition, the mobile unit is also equipped with common surgical kits and meets the requirement of surgery, so it can function as operating theatre when necessary. To our knowledge, this is the first research to study the feasibility of minimally invasive intervention angiography plus embolization to treat solid organ injuries in abdomen, such as splenic injury, in the mobile minimally invasive interventional shelter.

Injuries to the solid abdominal viscera occur frequently in natural disasters, and will be dangerous or fatal when hemorrhage occurs. Furthermore, the evacuation inconvenience in the disaster areas adds to the risk and leads to hemorrhagic shock, and even death. The mobile minimally invasive interventional shelter is able to offer first aid to patients with splenic injury and provide angiographic diagnosis and embolization treatment for the damaged vessels, thus preliminarily controlling the injury to buy time for evacuation and further treatment, and reducing both the death and disability rates of the casualty.

Some researchers have found that it has the advantage of both sensitivity and specificity to choose selective celiac arteriography as the diagnostic approach of hemorrhage caused by abdominal viscera injury [2]. It is also reported that artery embolization is effective in treating injuries to solid abdominal viscera without sacrificing their function [9]-[11].

The treatment for open splenic injury is well established. If the patients are hemodynamically stable, a simple debridement is adequate. However, if the hemodynamics of the wounded is unstable or simple compression hemostasis does not work, and when the on-spot operation conditions are unsatisfactory, the emergent debridement, arteriography diagnosis and interventional embolic hemostasis can be performed simultaneously in the mobile minimally invasive interventional shelter as fast as possible, which can facilitate evacuation and further treatment.

The results of contrast-enhanced CT are still the gold standard in diagnosing injuries to solid abdominal viscera [12],[13]. According to American Association for the Surgery of Trauma (AAST) grading system, the patients with Grade I and II splenic injury should lie in bed without any surgery treatment. Empirically, selective arteriography and embolization can serve as an alternative to the treatment of Grade III and IV splenic injury as well as Grade V splenic injury when hemodynamics is unstable [14]-[16]. Yet in natural disasters, CT scan apparatus is rarely accessible. Therefore, ultrasonic inspection, abdominal paracentesis, clinical symptoms and signs can be considered as substitutes in diagnosis and clinical classification of the injuries in disaster areas. In some trauma recovery centers in Europe, focused abdominal sonography for trauma (FAST) system based on B-ultrasonic inspection is still in use to rapidly diagnose hemorrhage caused by fatal injuries to solid abdominal viscera [17]. Many researchers report that B-ultrasonic inspection for quick diagnosis of abdominal trauma can be performed simultaneously with resuscitation during initial trauma management, and it only takes less than 2 min to complete rapid classification of a patient with unstable hemodynamics, who is receiving on-going cardio-pulmonary resuscitation [18],[19]. Most trauma centers that use FAST as a primary screening modality rely on the assumption that any missed injuries are low-grade lesions without serious clinical consequence [20],[21]. The mobile minimally invasive interventional shelter is equipped with portable B-ultrasonic apparatuses, which is available in the natural disaster areas to rapidly diagnose intra-abdominal hemorrhage, including that caused by splenic injury. A positive result may accelerate the angiography and embolization to control or heal injury. For patients with stable hemodynamics or negative ultrasonic result, further observation and evacuation are reasonable.

Although arterial embolization has been widely applied in patients with closed abdominal injuries [15],[16],[22], surgery is still a main choice to stop the bleeding caused by open abdominal injury. There is a lack of study focusing on on-spot emergent interventional embolization treatment of open abdominal injury. As for a fetal hemorrhage caused by puncture or shrapnel wounds, embolization is highly advisable before emergent evacuation, especially when the poor condition makes an operation impossible during natural disasters, catastrophes or other emergencies. On-spot emergent interventional embolization treatment should also gain valuable time for evacuation and further treatment, thus reducing the death rate.

It is well recognized that enough attention should be paid to preserving the spleen in managing traumatic splenic injury. Interventional splenic artery embolization treatment is now a minimally-invasive, convenient, efficient and reliable hemostasis method [23],[24]. It not only reduces the spleen artery pressure, but also enables the spleen to get sufficient blood supply by establishing collateral circulation with arteriae gastricae breves and gastroepiploic artery [25]. Thus the function of the spleen can be well preserved. Besides, it saves the sources of blood transfusion, reduces the complications and accelerates recovery process.

This minimally invasive interventional shelter is highly mobile, independent, and multifunctional. The shelter, which can be used either independently or as a part of the field hospital, is able to provide professional, immediate and minimally-invasive first aid during emergencies. This research simulates the treatment of patients with splenic injury in mobile minimally invasive interventional shelter in field environment. After the operation, we find no infection in the injured areas in the 8 animal models of splenic injury, and find only one nonfatal pseudoaneurysm. Above all, the advantages of the novel mobile minimally invasive interventional shelter are as follows: (1) it is safe and efficient to perform on-spot emergent interventional embolization in patients with severe parenchyma organ injury in the shelter, which stanches bleeding without removing organs; (2) the mobile interventional shelter can be adapted to the field environment perfectly, shorten the interval between the injury and treatment dramatically, reduce mischance during emergent evacuation, gain more time for further treatment, and finally save the patients’ life and improve prognosis; (3) the mobile unit brings minimally invasive interventional therapy from catheterization laboratory of top hospitals to areas of interest outside hospitals (such as disaster areas) and improves the poor treatment conditions following natural disasters greatly; (4) a new model of treating patients with severe parenchyma organ injury in exceptional environments may be established based on the mobile unit, and more lives will be saved.

However, there are still some limitations of the mobile minimally invasive interventional shelter and this research: (1) the medical materials reserved in the mobile are limited and external support is needed when the medical staff go out to perform rescue mission for a long time; (2) A fully functional recovery shelter is needed to monitor postoperative patients until their condition is stable to avoid the risk of long-distance transfer to base hospitals after interventional operation. (3) This research is performed in the environment that simulates disasters; thus further clinical studies are needed to determine the utility of the mobile unit for the treatment of severe parenchyma organ injury during medical rescue in real disasters.

## Conclusions

In the current study, we demonstrate that the mobile minimally invasive interventional shelter can be adapted to the field environment perfectly and perform emergency surgical intervention for splenic injury efficiently and safely. On-spot emergent transcatheter arterial embolization for vascular injury to solid organs (for instance, spleen) in the mobile minimally invasive interventional shelter is feasible and effective. This kind of shelter may enhance the on-spot therapeutic effects of injuries to solid abdominal viscera in some special cases, such as in the event of a natural disaster.

## Abbreviations

TAE: Transcatheter arterial embolization

UV: Ultraviolet

ECG: Electrocardiograph

CT: Computed tomography

AAST: American association for the surgery of trauma

FAST: Focused abdominal sonography for trauma

## Competing interests

There are no competing interest and non-financial competing interests exist when we interpretation of data or presentation of information. And we have no any financial relationship with any industry which may bias the outcome of this study. This work was supported by the Key Project of “the Twelfth Five-Year Plan” for Medical Science and Technology Research of People’s Liberation Army (No. BWS12J006). This work was also supported by Doctor Startup Fund of Liaoning Province (NO. 20131135) and Youth Foundation of Medical Science and Technology Research of People’s Liberation Army (NO.13QNP003).

## Authors’ contributions

YH conceived of the study, and participated in its design and helped to draft the manuscript. TY and ML designed and coordinated the study. TY, JR, ML, JS, FX, XW, FL and GW carried out the experiments. TY and JR drafted the manuscript. All authors read and approved the final manuscript.

## Authors' information

YT, Associate Chief Physician of Cardiology in Shenyang Northern Hospital, Doctor of Cardiology.

JR, Attending Physician of Cardiology in Shenyang Northern Hospital, Doctor of Cardiology.

ML, Associate Chief Physician of Cardiology in Shenyang Northern Hospital, Doctor of Cardiology.

JS, Senior Engineer of Medical engineering section in Shenyang Northern Hospital, Doctor of Engineering.

FX, Resident Physician of Cardiology in Shenyang Northern Hospital, Master of Cardiology.

XW, Chief Physician of Cardiology in Shenyang Northern Hospital, Doctor of Cardiology.

FL, Associate Chief Nurse of Cardiology in Shenyang Northern Hospital.

GW, Associate Chief Physician of Cardiology in Shenyang Northern Hospital, Doctor of Cardiology.

YH, Chief Physician of Cardiology in Shenyang Northern Hospital, Doctor of Cardiology.
